# Repetitive Transcranial Magnetic Stimulation Therapy (rTMS) for Endometriosis Patients with Refractory Pelvic Chronic Pain: A Pilot Study

**DOI:** 10.3390/jcm8040508

**Published:** 2019-04-13

**Authors:** Anne Pinot-Monange, Xavier Moisset, Pauline Chauvet, Anne-Sophie Gremeau, Aurélie Comptour, Michel Canis, Bruno Pereira, Nicolas Bourdel

**Affiliations:** 1 Department of Gynecological Surgery, CHU Clermont-Ferrand, 63000 Clermont-Ferrand, France; annepinot63@gmail.com (A.P.-M.); pchauvet@chu-clermontferrand.fr (P.C.); asgremeau@chu-clermontferrand.fr (A.-S.G.); acomptour@chu-clermontferrand.fr (A.C.); mcanis@chu-clermontferrand.fr (M.C.); nbourdel@chu-clermontferrand.fr (N.B.); 2 Department of Neurology, CHU Clermont-Ferrand, 63000 Clermont-Ferrand, France; 3 Neuro-Dol, University of Clermont Auvergne, Inserm U1107, 63000 Clermont-Ferrand, France; 4 Biostatistics Division (DRCI), CHU Clermont-Ferrand, 63000 Clermont-Ferrand, France; bpereira@chu-clermontferrand.fr

**Keywords:** endometriosis, pain, transcranial magnetic stimulation

## Abstract

Endometriosis concerns more than 10% of women of reproductive age, frequently leading to chronic pelvic pain. Repetitive transcranial magnetic stimulation (rTMS) over the primary motor cortex (M1) induces an analgesic effect. This effect on chronic pelvic pain is yet to be evaluated. The objective of this study was to assess the feasibility and effect of rTMS to reduce pain and improve quality of life (QoL) in patients with chronic pelvic pain due to endometriosis. This pilot, open-labelled prospective trial examined treatment by neuronavigated rTMS over M1, one session per day for 5 consecutive days. Each session consisted of 1.500 pulses at 10 Hz. We assessed tolerance, pain change and QoL until 4 weeks post treatment with a primary endpoint at day 8. Twelve women were included. No patients experienced serious adverse effects or a significant increase in pain. Nine women reported improvement on the Patient Global Impression of Change with a reduction in both pain intensity and pain interference (5.1 ± 1.4 vs. 4.1 ± 1.6, *p* = 0.01 and 6.2 ± 2.1 vs. 4.2 ± 1.5, *p* = 0.004, respectively). rTMS appears well tolerated and might be of interest for patients suffering from chronic pelvic pain for whom other treatments have failed. A randomized controlled trial is mandatory before proposing such treatment.

## 1. Introduction

Endometriosis is a very frequent pathology, concerning 10% of women of reproductive age and at least 20% of women with pelvic pain [1,2]. Pain is the leading and one of the most debilitating symptoms in this pathology. Though many clinicians and patients attribute endometriosis-associated pain to lesions, causality remains to be established. As endometriotic lesions are able to develop their own nerve supply thereby creating a direct interaction between lesions and the nervous system, pain can in some women, become independent of the disease itself [3]. Hormonal treatment for pain works by suppressing ovulation and thinning the endometrium and results in lighter menses. This can influence the sensitivity of peripheral sensory neurons as well as sympathetic nerve actions on those neurons and so modulate central neuronal activity in pain states [3]. Surgery is another step in the treatment process but may in some cases fail to be fully effective concerning pain. Disease recurrence or postoperative fibrosis offer a partial explanation for the persistence or recurrence of pain, while central or peripheral sensitization are also likely involved. Research in its early stages has focused on endometriosis pain pathophysiology, point to pain being nociceptive, neuropathic or a combination of both. Numerous painkillers have also been tested with variable results (non-steroidal anti-inflammatory treatment, morphine, treatment for neuropathic pain) and patients may benefit from physiotherapy or alternative medicines [4,5]. Even with combined therapeutic approaches not all patients experience pain relief, for many of whom chronic intractable pain is a major problem despite repeated surgery. This condition is frequently associated with medication overuse, impairment of quality of life and depression [6].

Repetitive transcranial magnetic stimulation (rTMS) has been proven to be effective for various pain types, nociceptive, neuropathic and even nociplastic [7,8,9,10,11], however, data concerning rTMS efficiency in chronic visceral pain is very limited [12,13]. It is important to note that access to this technique for chronic pain treatment is still limited worldwide. Nonetheless, in Europe, several rTMS devices have obtained Conformité Européenne (CE) Mark and are applied clinically for multiple neurological disorders including pain.

The aim of our pilot study was to assess the feasibility, safety and potential efficacy of rTMS treatment for refractory pelvic chronic pain due to endometriosis.

## 2. Materials and Methods

### 2.1. Ethics Approval

The protocol ENDOSTIM is a registered Clinical Trial (NCT03204682) and received approval from the Southeast Committee for the Protection of Persons VI (AU 1219; 06/02/2016). 

### 2.2. Design of The Study

We performed an open-labelled, prospective trial evaluating the feasibility and tolerance of five rTMS sessions over 5 consecutive days in endometriosis patients with chronic pelvic pain.

### 2.3. Patient Selection

Inclusion criteria included patients with histologically confirmed endometriosis experiencing at least one painful symptom related to endometriosis (dyspareunia, dysmenorrhea, pain with defecation or urination or neuropathic pain) with an average numeric rating scale > 4/10 for at least 4 days out of 7 for at least 3 months, continued hormonal treatment failure, adequate oral and written French, and the possibility of monitoring for the duration of the study (4 weeks). Patients were experiencing failure of both drug and surgical treatment. Written informed consent was obtained from each patient. Exclusion criteria included: prior treatment with rTMS, contra-indication to rTMS, another more severe pain than that associated with endometriosis, patients under guardianship, or those unable to understand informed consent.

### 2.4. Evaluation Criteria

**Figure 1:**
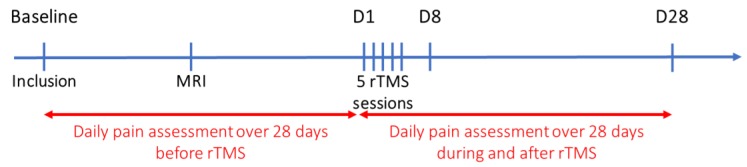
Overview of the protocol. rTMS: Repetitive transcranial magnetic stimulation; D1: day 1; D8: day 8; D28: day 28. MRI: magnetic resonance imaging.

Once enrolled, a clinical evaluation of symptoms associated with endometriosis (physical examination and surveys) was performed. Patients completed an electronic “daily pain” survey for 28 days before the first rTMS session and a second “daily pain” survey for 28 days after this session, in order to evaluate response to treatment (Figure 1).

The primary outcome involved the percentage of patients undergoing the five rTMS sessions, without serious adverse effects and without significant increase in pain at day 8 (D8) when compared to the pain level assessed over the previous 4 weeks. A variation of 10% was considered as significant [14]. Patients were asked to report any adverse effects of stimulation at each visit.

Secondary outcomes took into account several measures of chronic pain caused by endometriosis. Firstly, the variation between the mean pain before inclusion and day 8 (D8, day 1 referring to the first rTMS session) was measured using a visual analogue scale with a range from 0 to 100. The next variations in survey scores were analyzed before stimulation and at D8 and D28 (Endometriosis Health Profile Questionnaire (EPH-30) [15]) with Brief Pain Inventory (BPI) [16]; Patient Global Impression of Change (PGIC) measure; the State-Trait Anxiety Inventory [17]; Medical Outcomes Study Short Form 36 (SF-36) Quality of Life Questionnaire [18]; Intestinal gastrointestinal quality of Life Index (GIQLI) [19]) with the aim of evaluating the emotional impact of pain and any improvement in quality of life. Evaluation of the GIQLI score was carried out in accordance with previous validation [19]: The higher the score, the better the quality of life, with a maximum total score of 144 and an average score for healthy subjects of 126. For EHP-30 results, lower scores correspond to a better health status [15].

Other emotional aspects of chronic pain were evaluated from responses to the following questionnaires: The Beck Questionnaire (Depression) [20]; The State-Trait Anxiety Inventory (STAI); The Pain Catastrophizing Scale [21]; and the French version of the 20-items Toronto Alexythymia Scale [22]. 

### 2.5. Experimental Procedure

Each volunteer underwent brain MRI (3T, 3D T1-weighted axial and functional MRI of right hand motor cortex activation) prior to the first stimulation session to rule out brain abnormalities and to facilitate the neuronavigation procedure.

Magnetic stimulation was applied by way of a MagProX100 machine (Magventure Tonika Elektronic, Denmark) using a B65 figure-of-eight-shaped coil oriented tangentially to the scalp in the anterior–posterior direction. This coil was fixed to an arm that could be adjusted in three dimensions. The coil was positioned over the left M1 (primary motor cortex) with an optical neuronavigation device (Kolibri, Brainlab). We chose to target the left M1 over the hand representation as in previous studies [7,23]. Indeed, there is no clear somatotopy for pain treatment [24,25] and such unilateral stimulation has shown to be effective to treat diffuse pain.

The resting motor threshold (RMT) was defined as the lowest intensity eliciting a motor evoked potential (MEP) with a peak-to-peak amplitude of at least 50 mV in 50% of trials. MEPs were recorded for the first interosseous muscle of the right hand, with an electromyogram amplifier module (Magventure Tonika Elektronic, Denmark) and surface electrodes, as in previous studies [26,27].

A stimulation session consisted of 15 10-s pulses at 10 Hz, separated by a 50-s interval, for a total of 1500 pulses and applied at 80% of the RMT [7,26,27,28].

### 2.6. Statistical Analyses

This pilot study allowed assessment of the feasibility and safety of rTMS treatment for refractory endometriosis pain, paving the way for a future randomized controlled trial. The sample size estimation was fixed in accordance with Fleming’s multi-stage design, to include one group and multi-stages (between 1 and 5) providing filtering steps leading to a decision type go/no go. In view of capacity and feasibility, two steps were maintained with a lower boundary of 25% (maximum inefficacy) and an upper boundary of 50% (minimum efficacy) as recommended in the literature. Due to the type of design and hypotheses, type I error α and statistical power corresponded respectively to 5% and 80% with the following decision rules: With 12 patients included at the end of the first stage, treatment was considered not feasible if three or more patients reported a pain increase or did not tolerate the five rTMS sessions, and feasible if seven or more responses were observed. In the event of four to six responses being observed, 12 additional patients would be needed to make definitive conclusions with a feasible treatment requiring 10 or more responses.

The statistical analysis was performed using Stata software, version 13 (StataCorp, College Station, TX, USA). The tests were two-sided, with type I error set at α = 0.05. Continuous data were presented as the mean ± standard deviation or the median [interquartile range] according to statistical distribution (assumption of normality checked using normal probability plots and Shapiro-Wilk’s test). The percentage of patients tolerating five stimulation sessions and showing no pain increase on D8, compared to assessment performed over the previous 4 weeks, was presented with a 95% confidence interval. Paired comparisons were carried out using the Student paired t-test or Wilcoxon test. Effect size for repeated measures is estimated using the difference between two means divided by a standard deviation and taking into account within-patient correlation [29]. Effect size can be analyzed according to the recommendations of Cohen [30] who defined effect-size (ES) bounds as: small (ES: 0.2), medium (ES: 0.5), or large (ES: 0.8, “grossly perceptible and therefore large”). Repeated measures were analyzed using random-effects models taking into account between and within patient variability (patient as random-effect). Comparisons were carried out applying a type I error correction due to multiple comparisons (Sidak’s correction).

The normality of residuals was studied using Shapiro-Wilk’s test. When appropriate, a logarithmic transformation was proposed so as to achieve normality for the dependent outcome.

### 2.7. Ethical and Legal Considerations

The study was registered (NCT03204682) and received the ethic committee’s approval (Comité de Protection des Personnes Sud-Est 06).

## 3. Results

Thirteen women with chronic pelvic pain due to endometriosis were included from April 2016 to August 2017. One patient withdrew from the study before the first rTMS session due to fear of possible side effects and another patient included in accordance with the Fleming’s design. Thus, 12 patients completed the study (Table 1). The mean chronic pain duration was 9 years.

### 3.1. Feasability

The main outcome was achieved with a tolerance rate of 100% (All patients tolerated the five rTMS sessions and none reported a significant pain increase). No serious adverse effects were noted, however, 50% of patients reported experiencing light headaches and 25% described asthenia. 

### 3.2. Impact on Pain

**Figure 2:**
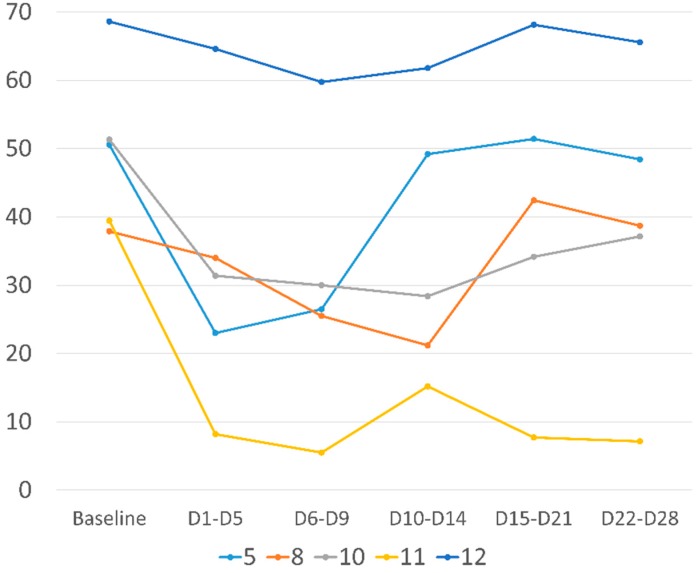
Evolution of daily pain over time for the five patients with more than 10% reduction at day 8 (D8), day 1 corresponding to the first rTMS session. Pain is noted out of 100.

Five patients experienced a daily VAS improvement of at least 10% between the 28 days before treatment and day 8 after rTMS, four of them having experienced an improvement of more than 30% (Table 1 + Figure 2). Data obtained from the Brief Pain Inventory, between pre-treatment and D8 revealed a significant improvement in the overall intensity of pain (5.1 ± 1.4 vs. 4.1 ± 1.6, *p* = 0.01; ES = 0.74 (0.17; 1.30)) and in the overall impact of pain on quality of life (6.2 ± 2.1 vs. 4.2 ± 1.5, *p* = 0.004; ES = 0.85 (0.27; 1.41)). These significant improvements were found to continue until D28 for both intensity (5.1 ± 1.4 vs. 4.3 ± 1.9; *p* = 0.048; ES = 0.57 (0.01; 1.13)) and interference (6.2 ± 2.1 to 4.6 ± 3.0; *p* = 0.02; ES = 0.68 (0.12; 1.26)) (Table 2). 

### 3.3. Patient Global Impression of Change and Quality of Life

PGIC results on D8 showed that nine patients experienced some improvement (eight minimally and one much improved) (Table 1). Four of the minimally improved patients had no significant variation in pain. 

**Figure 3:**
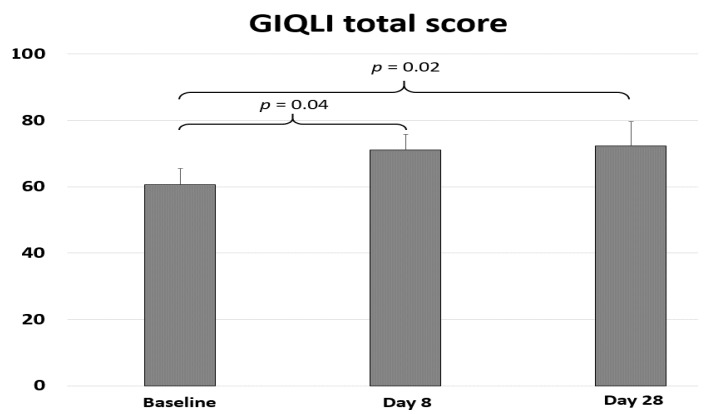
Evolution of gastrointestinal quality of life (GIQLI) scores before rTMS (baseline) and at day 8 and day 28; day 1 corresponding to the first rTMS session.

Concerning gastrointestinal quality of life, a significant improvement was observed after treatment (*p* = 0.04; ES = 0.60 (0.04; 1.17)), with a total GIQLI (gastointestinal quality of life) score of 60.6 ± 14.0 before stimulation and 71.1 ± 13.3 at D8. Continued significant improvement was noted until D28 with a score of 72.4 ± 20.8 (*p* = 0.02; ES = 0.68 (0.10; 1.26)) (Figure 3). This GIQLI variation was not significantly correlated with pain intensity variation (r = 0.10, *p* > 0.05). The GIQLI score is made of sub-scores focusing on symptoms, emotions, physical function, and social function. The most important variation was observed for symptoms, the sub-score going from 37.8 ± 8.7at baseline to 44.3 ± 9.4 at day 8 (*p* = 0.002; ES = 0.90 (0.33; 1.47)). The other sub-scores did not present a statistically significant variation. 

Assessment by SF-36 at D8 showed that the improvement in Quality of Life was marginally significant for the physical component (37.5 ± 7.9 vs. 42.0 ± 9.7; *p* = 0.047; ES = 0.88 (0.01; 1.75)) and unchanged for the mental component (36.8 ± 10.6 vs. 36.8 ± 10.2; *p* = 0.77; ES = 0.1 (−0.74; 0.94)). 

Following rTMS treatment (D8), several changes in EHP-30 questionnaire sub-scores were noted, notably an improvement in emotional well-being (54.9 ± 15.5 vs. 43.1 ± 19.6; *p* = 0.02; ES = 0.86 (0.02; 1.70)) together with a non-significant improvement in pain levels (65.0 ± 16.3 vs. 57.6 ± 15.7; *p* = 0.06; ES = 0.58 (−0.23; 1.40)). There was a significant correlation between pain variation on day 8 and the variation of the emotional part of the EHP30 questionnaire (r = 0.76, *p* < 0.05).

At baseline, 91.7% (11/12) of women reported a depressive state according to Beck’s questionnaire results and 50% were alexithymic. STAI at D8 revealed no variation in anxiety compared to pretreatment scores (48.3 ± 5.0 to 45.2 ± 5.1, *p* = 0.30; ES = 0.62 (−1.48; 0.24)).

## 4. Discussion

Our study demonstrates a perfect tolerance of five daily 10 Hz rTMS sessions in patients with chronic refractory pelvic pain due to endometriosis. Overall, pain intensity was reduced by 1 point (5.1 ± 1.4 vs. 4.1 ± 1.6, *p* = 0.01; ES = 0.74 (0.17; 1.30)) and pain interference by 2 points (6.2 ± 2.1 vs. 4.2 ± 1.5, *p* = 0.004; ES = 0.85 (0.27; 1.41)), with moderate to large effect sizes. PGIC assessment revealed an improvement for a large majority (9/12) of these patients experiencing refractory pain. An improvement was also noted in the physical component of the SF-36 and for gastro-intestinal quality of life parameters.

In the present study, PGIC did not perfectly correlate with pain reduction, highlighting that reducing pain is not the only way to improve patient quality of life. In addition, gastrointestinal quality of life improvement was poorly correlated with pain reduction. It has been shown that repeated sessions of high-frequency motor cortex rTMS delivered on the bihemispheric cortical representation of the lower limb muscles is able to improve daily functioning in patients with chronic neuropathic pain affecting the lower limbs, even in the absence of pain relief [31]. Such a “paradox” has also been shown in fibromyalgia patients who reported improvements in quality of life in the absence of significant pain reduction [8]. Our results together with these observations, suggest that the action on certain brain circuits during motor cortex rTMS may have a therapeutic impact on refractory chronic pain patients and that the various induced changes can contribute to long-term patient satisfaction, even if pain is not drastically reduced.

Although statistically significant, the pain intensity reduction of 1 point (20%) in this open-label study is low. A recent meta-analysis defined a minimal clinically important difference (MCID) of 10 mm using the VAS for women with pain due to endometriosis [14]. For BPI, MCID has never been evaluated specifically in endometriosis. In other chronic pain conditions, MCID has been estimated around 2 points and around 30% or with an effect size of 0.5 [32,33,34]. In the present study, ES is above 0.5 for both pain intensity and interference but the 2 points and 30% thresholds are reached for pain interference only. Again, this result suggests that pain impact can be reduced more than pain intensity using rTMS in this condition. As shown in Figure 2, pain relief is not very long-lasting after a single set of rTMS sessions. Concerning fibromyalgia and chronic neuropathic pain, it has been shown that maintenance sessions were necessary to maintain pain relief [23,35]. Such sessions can be spaced progressively to 3 weeks and a cumulative effect has been suggested, with stronger pain relief and longer pain relief duration after each session when continuing rTMS session for several months [35]. 

Concerning quality of life, it has been demonstrated that SF-36 and EHP-30 perform well in the evaluation of endometriosis patients [36]. In the present study, the physical component of the SF-36 and the emotional component of the EHP-30 were improved. The improvement concerning the emotional component of the EHP-30 was correlated with pain intensity variation. Our results revealed an 11-point improvement in gastrointestinal quality of life. This is of particular interest, as to date, to our knowledge, no other study has shown such an improvement following non-invasive treatment in endometriosis. The GIQLI questionnaire used to evaluate this parameter has undergone thorough validation, with MCID considered to be between 6 and 8 points [37].

This study is the first to study rTMS in the management of endometriosis using a protocol proven effective for neuropathic pain and fibromyalgia [9,10] and neuronavigation for optimal precision [38]. Currently, only two studies have investigated the effects of rTMS on visceral pain. The first focused on irritable bowel syndrome and used the same rTMS protocol as in the present study [13]. Their results showed that patients who benefited from real rTMS experienced a significant improvement in rectal tolerated volume with a non-significant improvement in mean daily pain. The second reported a significant reduction in pain due to chronic pancreatitis after daily rTMS sessions over 10 days [12]. The rTMS protocol was however very different as 1600 pulses at 1 Hz were delivered over the right secondary somatosensory cortex.

To date, high frequency (i.e., more than 5 Hz) rTMS over M1 is the only validated protocol for pain treatment [9,10,39]. Although epidural motor cortex stimulation seems more effective if the motor representation of the painful area is stimulated [40], such somatotopy is not obvious using rTMS [24,25]. Although the link between M1 stimulation and pain relief is not obvious at first, M1 can be seen as an entry door into a larger network involved in pain modulation [41]. Concerning other non-invasive brain stimulation techniques for abdominal pain treatment, there is some evidence for the efficacy of anodal transcranial direct current stimulation (tDCS) over the primary motor cortex and recent guidelines provided a level B for its use in such pain situation [39]. In the case of rTMS, other possible brain targets for pain relief are under investigation, especially the dorsolateral prefrontal cortex (DLPFC) or deeper cortical areas [40]. Repetitive TMS is approved for the treatment of depression and is highly effective [9,42]. When used for the treatment of depression, the targeted cortical area is different (DLPFC). For pain treatment, the primary motor cortex is targeted and such stimulation is not effective in depression treatment [10,39].

The main limitations of the present study concern the absence of a control group and the limited sample size. The placebo effect can be important in pain studies, though likely to be limited in patients with chronic pain undergoing rTMS [43]. Using Fleming’s multi-stage design, inclusion was stopped at 12 patients, in accordance with the primary criteria for feasibility. A future randomized control trial that enrolled 80 patients with chronic pelvic pain would allow to show a 30% difference in the proportion of patients with at least minimal improvement on the PGIC scale (30% in the placebo group vs. 60% with active rTMS), using a type 1 error of 5% and a power of 80%. The present study also has a limited follow-up period. Future trials should aim to report on patient improved quality of life for a least 12 weeks, using maintenance rTMS sessions, as proposed in the literature [23,31,40,44].

## 5. Conclusions

This prospective pilot study showed that high frequency motor rTMS is feasible and well-tolerated in patients with refractory chronic pelvic pain due to endometriosis. A majority of these patients reported at least minimal improvement on the PGIC scale after five rTMS sessions. Although pain reduction was modest, the impact on pain interference and gastro-intestinal quality of life was perceived as beneficial for these women. This study paves the way for a future study of analgesic efficacy using a double-blind trial and presents initial promising support for a new analgesic technique for use in daily clinical practice in patients for whom all other treatments have failed. Although non-invasive and well-tolerated, a confirmatory study using a double-blind design is mandatory before developing such treatment in daily practice.

## Figures and Tables

**Table 1:** Patients’ characteristics. Mean values are noted using bold characters.

	Age	Pain Treatment	Mean Baseline Pain	Pain at D8	Pain Variation	PGIC at D8	Pain at D28
1	49	Tramadol	53	57	+8%	Minimally improved	27
2	32	No	54	50	−7%	No change	60
3	41	NSAID, tramadol	40	40	+1%	No change	53
4	34	No	70	75	+7%	No change	74
5	26	Morphine	51	4	−92%	Much improved	69
6	48	Acetaminophene	54	53	−3%	Minimally improved	53
7	32	Mephenesine	50	50	0%	Minimally improved	35
8	39	No	38	21	−45%	Minimally improved	27
9	52	NSAID	55	50	−10%	Minimally improved	50
10	29	NSAID	51	25	−51%	Minimally improved	40
11	40	No	39	3	−92%	Minimally improved	5
12	40	NSAID, Acetaminophene	69	58	−15%	Minimally improved	63
Mean	38 ± 8		50 ± 13	41 ± 22	−18%	9/12 improved	46 ± 16

Mean baseline pain correspond to the mean pain completed daily on the pain diary for the 28 days before first rTMS session. PGIC = patient global impression of change; rTMS: Repetitive transcranial magnetic stimulation; D8: day 8. NSAID: non-steroidal anti-inflammatory drug.

**Table 2:** Brief pain inventory (pain intensity and pain interference) at day 1, day 8 and day 28. Mean values are noted using bold characters.

	Intensity D1	Intensity D8	Intensity D28	Interference D1	Interference D8	Interference D28
1	5.3	4.8	3.8	8.0	2.3	2.6
2	5.0	3.8	6.5	8.6	3.6	8.9
3	7.0	6.3	5.8	7.9	7.1	6.3
4	7.0	4.5	2.5	6.7	4.4	3.1
5	4.8	1.8	3.8	8.3	4.0	7.1
6	3.3	3.5	1.0	5.6	4.1	0.7
7	5.3	5.3	5.5	8.3	5.1	8.1
8	4.0	2.8	4.0	6.3	4.6	4.0
9	4.5	4.8	4.8	4.0	6.0	7.9
10	4.5	3.5	5.0	4.6	3.0	0.1
11	3.3	1.5	2.0	2.0	1.9	1.7
12	7.3	7.3	7.3	4.4	4.4	4.4
Mean	5.1 ± 1.4	4.1 ± 1.6	4.3 ± 1.9	6.2 ± 2.1	4.2 ± 1.5	4.6 ± 3.0

Patient 12 did not complete BPI (Brief Pain Inventory) at D8 (day 8) and D28 (day 28). Thus, baseline observation carried forward (BOCF) was applied.
